# Structural Model Identification Using a Modified Electromagnetism-Like Mechanism Algorithm

**DOI:** 10.3390/s20174789

**Published:** 2020-08-25

**Authors:** Zhouquan Feng, Zhengtao Ye, Wenzan Wang, Yang Lin, Zhengqing Chen, Xugang Hua

**Affiliations:** 1 Key Laboratory of Wind and Bridge Engineering of Hunan Province, College of Civil Engineering, Hunan University, Changsha 410082, China; det@hnu.edu.cn (Z.Y.); wenzanwang@hnu.edu.cn (W.W.); linyang@hnu.edu.cn (Y.L.); zqchen@hnu.edu.cn (Z.C.); cexghua@hnu.edu.cn (X.H.); 2 Vibration and Shock Technology Research Center, College of Civil Engineering, Hunan University, Changsha 410082, China; 3 State Key Laboratory of Advanced Design and Manufacturing for Vehicle Body, Hunan University, Changsha 410082, China

**Keywords:** structural model identification, electromagnetism-like mechanism, modal data, optimization

## Abstract

A modified electromagnetism-like mechanism (EM) algorithm is proposed to identify structural model parameters using modal data. EM is a heuristic algorithm, which utilizes an attraction–repulsion mechanism to move the sample points towards the optimal solution. In order to improve the performance of original algorithm, a new local search strategy, new charge and force calculation formulas, new particle movement and updating rules are proposed. The test results of benchmark functions show that the modified EM algorithm has better accuracy and faster convergence rate than the original EM algorithm and the particle swarm optimization (PSO) algorithm. In order to investigate the applicability of this approach in parameter identification of structural models, one numerical truss model and one experimental shear-building model are presented as illustrative examples. The identification results show that this approach can achieve remarkable parameter identification even in the case of large noise contamination and few measurements. The modified EM algorithm can also be used to solve other optimization problems.

## 1. Introduction

Modeling and identification of structural systems is a fundamental subject in structural engineering. In the past decades, structural system identification has been widely used in structural health monitoring, damage detection, response prediction, vibration control and other fields [1,2,3,4,5,6,7]. Structural system identification can be regarded as an optimization problem from the point of view of mathematics. Its goal is to find an optimal model and make its predictive response as close as possible to the measured response of the actual system [3]. According to the literature, the structural model can be divided into parametric physical model and nonparametric data model. The nonparametric model is completely based on data and sometimes it is called data-driven model, which is generally only used when the structural system is very complicated and the physical model is difficult to build [8]. Data-driven simulation models have been widely used in other fields, such as logistics facilities management [9] and multicriteria transportation problem [10]. Parametric structural models are generally defined by a set of physical parameters such as mass and stiffness, and they are usually modeled by the finite element method [11]. The process of solving the optimal parameters of the model is called parameter identification. The parameter identification of structural models is the foundation of all subsequent structural calculation and analysis.

Structural system identification can be divided into two types: frequency domain methods and time domain methods. The time-domain methods are directly based on the measured time-history data and represent the objective function as the residual between the predicted response and the measured data. The optimal model parameters can be obtained by solving this minimization optimization problem [3]. However, the response of the structural system is not only related to the structure itself but also related to the input of the system, so the input should also be measured or some assumptions should be made, which brings some inconvenience to the application. The frequency methods are based on the modal information of the structure, such as frequency response function and modal parameters [12]. In the identification of structural models based on modal parameters, the natural frequencies and mode shapes of the structure are first obtained through experimental modal analysis, and then the difference between the predicted modal parameters and the identified modal parameters is minimized by optimization method to estimate the model parameters.

Various techniques have been developed based on frequencies and mode shapes for structural identification. Brownjohn and Xia presented sensitivity-based model updating of a curved cable-stayed bridge based on measured modal data [13]. An iterative sensitivity-based FE model updating method was proposed by Teughels and De Roeck, in which the discrepancies in both the frequencies and mode shapes obtained from ambient tests are minimized [14]. Model updating based on frequency, mode shape, modal flexibility, and their combinations was studied by Jaishi and Ren [15]. Bakir et al. proposed a trust region algorithm for model updating to solve a nonlinear constrained optimization problem [16]. Adaptive regularization parameter optimization was proposed by Hua et al. for output-error-based FE model updating, in which an adaptive strategy was employed to change the value of the regularization parameter at different iteration steps [17]. Sarmadi et al. proposed a new iterative method named as least squares minimal residual for finite element model updating [18]. A tutorial was given by Mottershead et al. for the sensitivity-based finite element model updating with modal data [11].

However, most of the above methods generally need to obtain the gradient of the objective function and make a good initial guess of the solution. In addition, when measured data is incomplete, it may be difficult to use these methods in identifying large systems. Therefore, in recent years, heuristic optimization algorithms have been used to solve the optimization problem of structural model identification and achieved good results. Genetic algorithm was employed by Perera et al. for simultaneous optimizations of several objectives with the purpose of improving the performance of model updating [19]. Sun et al. proposed a modified artificial bee colony algorithm for structural identification [3]. A methodology based on particle swarm optimization with sequential niche technique was proposed by Shabbir and Omenzetter for dynamic finite element model updating [20]. Seyedpoor et al. proposed the differential evolution algorithm to identify multiple damage cases of structural systems [21]. Structural damage identification based on cuckoo search algorithm was presented by Xu et al. [22]. Performance studies of 10 metaheuristic techniques in structural model updating and damage detection were conducted by Mishra et al. [23].

In addition to the above heuristic algorithms, Birbil and Fang proposed a new heuristic algorithm called electromagnetism-like mechanism algorithm (EM) in 2003 to solve global optimization problems [24]. EM algorithm first produces a set of initial solutions (called a group of initial particles here) randomly from feasible domains, and then determines the attraction domain according to the objective function value of each particle, in order to produce a new generation of particles in some mechanism. The EM algorithm simulates the attraction and repulsion mechanism in the electromagnetic field, compares each solution to a charged particle, and then makes the particles move towards the optimal solution according to certain criteria. This idea comes from the analogy with the mechanism of attraction and repulsion in electromagnetic theory, which is called electromagnetism-like mechanism due to some differences between the two. More importantly, the convergence of EM algorithm has been proved, and the results show that at least one particle in the population moves near the global optimum with probability of one as the number of iterations is large enough [25]. However, the original version of EM algorithm has some defects to be improved, such as the charge and force formulas, the local search method and the particle movement rule. Several researchers have proposed some improvements to the original EM algorithm to enhance its performance [26,27,28,29]. Rocha and Fernandes proposed a modified EM algorithm based on a new local search method and a new movement force vector, in which a pattern search method replaced the simplest linear search, and a new vector is defined to consider information in both the current iteration and the previous iteration [26]. Zhang et al. proposed an improved electromagnetism-like mechanism algorithm for constrained optimization, in which four modifications are made to enhance its accuracy and efficiency [27]. Tan et al. improved the original EM algorithm by using a new local search scheme with Split, Probe and Compare feature (SPC-EM) [28]. Later, Tan et al. introduced an experience-learning feature into the EM particle, in which two new components including the memory concept and the experience analyzing and decision-making mechanism were employed [29]. The EM algorithm and its variants have been widely used in many application fields, such as project scheduling [30], vehicle routing [31], course timetabling [32], manufacturing system design [33], diabetes mellitus prediction [34], power flow control [35] and truss structure optimization [36,37]. However, there are very few studies related to structural engineering using EM algorithm [36,37]. These researches were limited to the optimal design of truss structures, and parameter identification of structural models was not involved. This study aims to improve the accuracy and computing efficiency by modifying the original EM algorithm. Then, the modified EM algorithm is applied for parameter identification of structural models.

The paper is organized as follows. In Section 2, structural system identification is described as an optimization problem. In Section 3, the original EM algorithm is reviewed and a modified EM algorithm is proposed for structural parameter identification. Section 4 tests the performance of the modified EM algorithm with benchmark functions and applies it in parameter identification of a numerical truss model and an experimental shear-building model. Some discussions are given in Section 5 for further study. In Section 6, we draw conclusions of this work.

## 2. Optimization Formulation of Structural Model Identification

Structural model identification can be regarded as the optimization problem of objective function. For example, the discrepancy between measured structural response and model prediction response is defined as the objective function, and parameter identification of the model is treated as optimization (usually minimization) of the objective function. If the data consist of several modal data sets, structural model parameter identification is to solve a set of optimal model parameters (e.g., denoted as $\theta \in {\mathbb{R}}^{N_{\theta }\times 1}$, *N_θ_* represents the number of parameters), so as to minimize the error between the predicted and measured modal parameters.

A linear structure model with *N* degrees-of-freedom (DOFs) is considered. The mass matrix ***M*** is assumed to be known and the stiffness matrix ***K*** is parameterized by ***θ***, namely, ***K*** = ***K***(***θ***). The global stiffness matrix ***K***(***θ***) can be represented as a cumulative sum of element matrices by using the following equation

(1) $$K\left(\theta \right)=\sum_{i=1}^{N_{e}}\left(1+\theta_{i}\right)ke_{i}$$

where *N*_e_ is the total number of finite elements and *ke*_i_ denotes the stiffness matrix of the *i*^th^ element. As we known that the predicted modal frequency ${\hat{f}}_{j}\left(\theta \right)$ and mode shape ${\hat{\phi }}_{j}\left(\theta \right)$ of *j*^th^ mode can be obtained through solving the following eigenvalue (characteristic) value problem.

(2) $$\left[K\left(\theta \right)-\omega^{2}M\right]\phi =0$$

where the quantities $\omega^{2}$ are the eigenvalues, which are the square of the circular modal frequencies (ω=2πf and *f* is the modal frequency in Hz), while the corresponding eigenvectors ϕ are the mode shapes.

The measured incomplete modal data are consisted of *N*_s_ sets of modal data (each data set contains modal frequencies and mode shapes with *N*_m_ modes). For a structural system with *N* DOFs, it should theoretically have *N* modes, and the mode shape of each mode contains *N* components. However, not all modes can be excited and some DOFs are difficult to be measured, therefore, the measured modal parameters are incomplete in practice. The data incompleteness is reflected in two aspects: one is measurement incompleteness, i.e., the number of measured DOFs *N*_o_ is less than *N* and the other is mode incompleteness, i.e., the number of identified modes *N*_m_ is less than *N*. Based on the least square principle, the sum of relative errors of modal data (modal frequencies and mode shapes) from prediction and measurement is used to establish the objective function. In this study, the objective function for model identification (updating) is defined as follows

(3) $$J\left(\theta \right)=\sum_{j=1}^{N_{m}}\left[\sum_{i=1}^{N_{s}}{\left(\frac{f_{j,i}-{\hat{f}}_{j}\left(\theta \right)}{f_{j,i}}\right)}^{2}+\sum_{i=1}^{N_{s}}\frac{\|\phi_{j,i}-a_{j,i}{\hat{\phi }}_{j}{\left(\theta \right)\|}^{2}}{\|\phi_{j,i}{\|}^{2}}\right]$$

where $a_{j,i}={\left(\phi_{j,i}\right)}^{T}{\hat{\phi }}_{j}\left(\theta \right)/\|{\hat{\phi }}_{j}{\left(\theta \right)\|}^{2}\;$ is a scaling factor that ensures that the predicted mode shape ${\hat{\phi }}_{j}\left(\theta \right)$ is closest to the measured mode shape $\phi_{j,i}$ at the measured DOFs [38], $f_{j,i}$ is the measured modal frequency of *j*^th^ mode in the *i*^th^ data set and ${\hat{f}}_{j}\left(\theta \right)$ is the predicted modal frequency of *j*^th^ mode, $\phi_{j,i}$ is the measured partial mode shape of *j*^th^ mode in the *i*^th^ data set and ${\hat{\phi }}_{j}\left(\theta \right)$ is the predicted partial mode shape of *j*^th^ mode.

## 3. Electromagnetism-Like Mechanism Algorithm

### 3.1. The Original EM Algorithm

**Figure 1:**
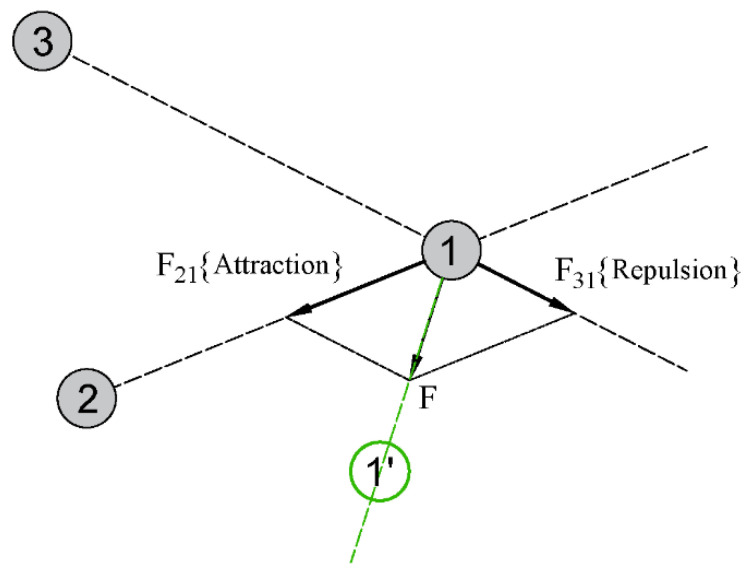
Schematic diagram of the mechanism of attraction and repulsion.

According to the attraction–repulsion mechanism in electromagnetic theory, EM algorithm imagines each solution as a charged particle in space, and the charge of each particle is determined by the value of the objective function to be optimized. This charge also determines how strongly the particle attracts or repels other particles: the better the objective function value, the stronger the attraction. The direction of each particle’s next move is determined by calculating the resultant force applied by other particles to the current particle. As with electromagnetic forces, the resultant force is obtained by superimposing vector quantities of forces from other particles. As shown in Figure 1, we select three particles to briefly illustrate how one particle searches according to the attraction–repulsion mechanism. Suppose that the solution represented by particle 2 is better than the solution represented by particle 1 and the solution represented by particle 3 is worse than the solution represented by particle 1, then particle 2 will have an attractive force *F*_21_ to particle 1 and particle 3 will have a repulsive force *F*_31_ to particle 1. The resultant force *F,* which is the superposition of two forces, will determine the direction in which particle 1 will move, forcing the particle to move towards a better region. Furthermore, similar to some other population-based hybrid algorithm, EM algorithm can also use a local search to improve the objective function value of the current population.

**Figure 2:**
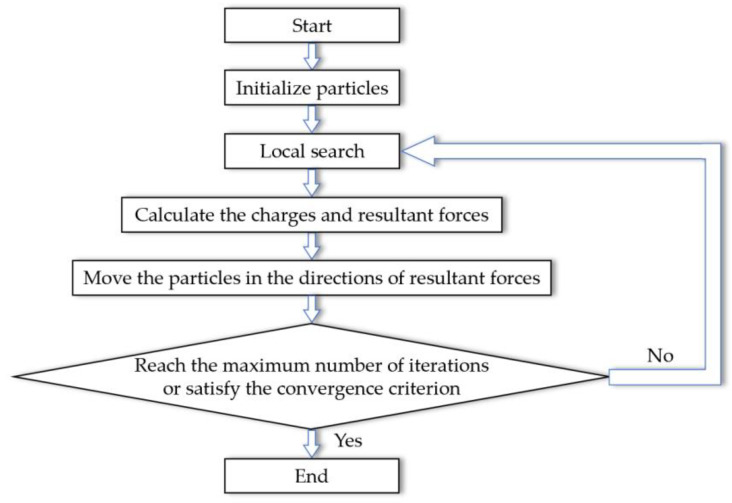
Flowchart of the original electromagnetism-like mechanism (EM) algorithm.

The EM algorithm consists of four steps, namely, initialization, local search, calculation of resultant force and movement of particles. The flow chart of the original EM algorithm is shown in Figure 2.

● Initialization

Initialization is to take a number of random points from a known feasible region. Here, the initial particles are randomly and uniformly distributed in the feasible region, then the objective function value of each particle is calculated and the particle with the optimal objective function value is denoted as $x_{best}$.

● Local search

Local search is performed on individual particles to improve the solution that the population has searched for. For EM algorithm, local search plays a very important role, because it provides effective local information for global search of population, so that the algorithm has both global search ability and fine search ability in local area. The local search used in the original EM algorithm is the simplest linear search, searching each dimension of each particle by a certain step size δ and stopping as soon as a better solution is found.

● Calculation of resultant force

The calculation of resultant force is the most important step of EM algorithm, which combines the local information obtained by particles with the global information. Simulating the superposition principle in electromagnetic theory, EM algorithm provides information for the next search by calculating the resultant force.

The charge quantity $q_{i}$ of particle *i* determines the magnitude of attractive force or repulsive force on particle *i*. The charge quantity $q_{i}$ is calculated as follows:

(4) $$q_{i}=exp\left(-n\frac{f\left(x_{i}\right)-f\left(x_{best}\right)}{\sum_{k=1}^{m}\left(f\left(x_{k}\right)-f\left(x_{best}\right)\right)}\right)$$

where f(x) is the objective function to be minimized, *n* is the dimension of the problem and *m* is the number of samples (particles) in each iteration.

In this way, particles with better objective function value will have larger charge value and stronger attraction. After comparing the objective function value of two particles, the direction of the interaction force between two particles will be determined. The resultant force *F_i_* acting on particle *i* is calculated as follows:

(5) $$F_{i}=\overset{m}{\underset{j\neq i}{\sum }}\left\{\begin{array}{c} \left(x_{j}-x_{i}\right)\frac{q_{i}q_{j}}{\|x_{j}-x_{i}\|{}^{2}}\;\;\;if\;f\left(x_{j}\right)<f\left(x_{i}\right)\\ \left(x_{i}-x_{j}\right)\frac{q_{i}q_{j}}{\|x_{j}-x_{i}\|{}^{2}}\;\;\;if\;f\left(x_{j}\right)\geq f\left(x_{i}\right) \end{array}\right\}$$

According to Equation (5), between every two particles, the particle with a better objective function value (i.e., the smaller one) will attract another particle. Conversely, a particle with a worse value of the objective function (i.e., a larger value) will repel another particle. Since the objective function value of the current optimal particle $x_{best}$ is the smallest, it acts as an absolutely attractive particle, attracting the whole population.

● Movement of particles

After calculating the resultant force vector *F_i_*, particle *i* will move along the direction of the resultant force with a random step length (as given in Equation (6)). The step *λ* is evenly distributed over [0, l]. In Equation (6), *RNG* is a vector whose *k*^th^ component represents the feasible step size towards the upper bound *u*_k_ or lower bound *l*_k_. In addition, the forces acting on each particle are “normalized,” thus ensuring the possibility of movement. The movement formula of the particle is

(6) $$x_{i}=x_{i}+\lambda \frac{F_{i}}{\left\|F_{i}\right\|}\left(RNG\right)\;\;\;i=1,2,\ldots ,m$$

In such a way, the position of the particles is updated and one iteration of the EM algorithm completes. The readers can see the reference for details of the original EM algorithm [24].

### 3.2. The Modified EM Algorithm

The EM algorithm is simple in structure, and it is not necessary to obtain derivatives of the function like many traditional methods to solve the global optimization problem. Moreover, the EM algorithm has no requirement for the type of function to be optimized. Therefore, this method can be used to solve global optimization of not only general functions but also of complex functions that are difficult to obtain information such as derivatives. However, in the original EM algorithm, the local search is the simplest random linear search. When the algorithm falls into a local minimum too early, it cannot jump out, which affects the optimization performance of the algorithm. Then, the calculation formulas for charge and force are not as good as possible, which affects the computation efficiency and precision. Next, the particle updating rules are fixed, and it is difficult to search the optimal value more accurately in the later stages of evolution, which affects the speed and accuracy of the algorithm. In order to solve the shortcomings of the original EM algorithm, we made improvements in the following five aspects to achieve better performance. These improvements are detailed below, and the pseudocodes for each modification are presented in the Appendix A.

● New local search method

**Figure 3:**
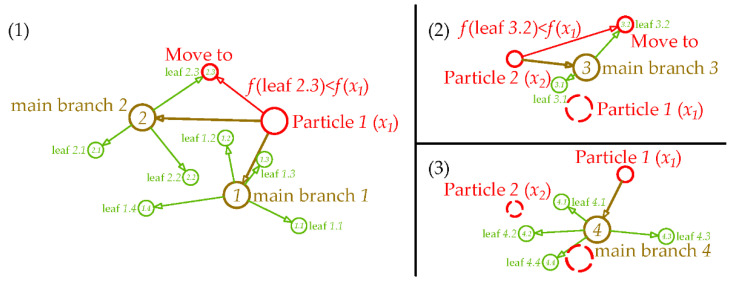
Graphical representation of the new local search method.

The original local search is the simplest linear search, which is not efficient. Here, we intensify local search by branching current best particles with a certain radius. For example, uniformly distributed points inside the ball centered at $x_{best}$ with radius of $\left\|x_{best}-x_{2^{nd}best}\right\|$ or $\left\|x_{best}-x_{3^{rd}best}\right\|$ are generated. Herein, $x_{best}$, $x_{2^{nd}best}$ and $x_{3^{rd}best}$ are the particle with best, second best and third best function value, respectively. Afterwards for each new point (main branches), what is done to $x_{best}$ to generate subpoints (leaves) is redone. Finally, all newly generated points to find if there is a point with better function value to replace current best particle are compared. The graphical representation of the new local search method is shown in Figure 3. Particle 1 is the best particle, and particle 2 is the second best particle. The red circles are the moving trail of particle 1 and particle 2, the brown circles are the main branches generated from the current best point and the green circles are the leaves generated from the main branches. In this rule, if particle 1 moves to a new point, particle 2 will move to the original position of particle 1. If the new point generated is not better than particle 1 but better than particle 2, particle 2 move to the new point without following particle 1. (Appendix A).

● Simplify charge calculation

The original charge formula with exponential calculations is complicated, and the function will become nearly linear in the interval [0,1]. In the later iterations of searching when many points crowd together, the exponents in the original charge formula is close to zero. Based on this observation, we propose to use a linear function to simplify the charge calculation. Some other researchers used linear functions for charge calculation as well, but in different forms [30,31]. The charge of particle used in the proposed modified EM algorithm is calculated as follows. (Appendix A)

(7) $$q_{i}=\frac{f\left(x_{i}\right)-\underset{j}{\min}\left\{f\left(x_{j}\right)\right\}}{\underset{j}{\max}\left\{f\left(x_{j}\right)\right\}-\underset{j}{\min}\left\{f\left(x_{j}\right)\right\}}$$

● ort particles and move particles with a new rule

**Figure 4:**
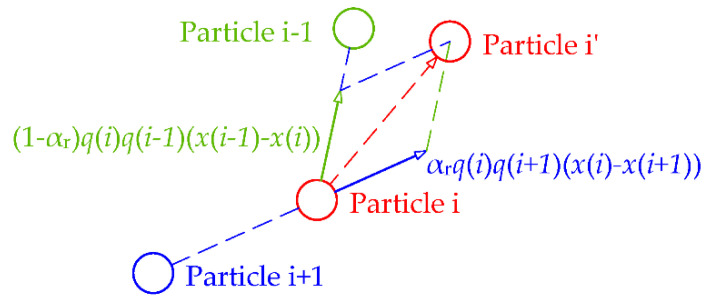
Graphical representation of new movement rule.

In the original algorithm, movement of particles does not care about how strong the total force is, and only the information of direction is made use of. If the magnitude of force is not necessary, one may come up with why the force formula needs to be in the form of Coulomb’s law. Therefore, it is perceived that the kernel of this optimizing algorithm is attraction of the better particles and repulsion of the worse. Inspired by a hybrid method combining electromagnetism-like mechanism and firefly algorithms [36], the proposed algorithm first have particles sorted then move them with only the attraction of one better particle and the repulsion of the next worse particle. In each iteration, the particles are sorted in ascending order according to their function values (Appendix A). Then, for particle *i*, it is attracted merely by particle *i*-1 and repelled only by particle *i*+1. The information of direction and charge decide where the particle will go, together with an adaptive rate of repulsion $\alpha_{r}$ that is uniformly distributed in [0, $\alpha_{u}$]. In the early iterations, the upper bound $\alpha_{u}$ is close to 1, emphasizing the repulsion to disperse particles in order of exploration. Diminish $\alpha_{u}$ as the number of iterations grows, shifting focus from exploration to exploitation. The graphical representation of the new movement rule is shown in Figure 4.

The attraction and repulsion forces of a sorted particle are defined as follows:

(8) $$F_{a}=\left(x_{i-1}-x_{i}\right)q_{i-1}q_{i}$$

(9) $$F_{r}=\left(x_{i}-x_{i+1}\right)q_{i}q_{i+1}$$

The movement is defined as follows:

(10) $$x_{i}^{’}=x_{i}+v_{EM}$$

(11) $$v_{EM}=F_{a}\left(1-\alpha_{r}\right)+F_{r}\times \alpha_{r}$$

where *Fa* and *Fr* are, respectively, attraction and repulsion forces acting on the particle *i* and $\alpha_{r}$ is a random control parameter that defines the electromagnetic behavior, and it is uniformly distributed in a range of [0,$\;\alpha_{u}$]. $\alpha_{u}$ is the upper bound of the $\alpha_{r}$, which changes its value with the number of iterations as follows. (Appendix A)

(12) $$\alpha_{u}=\;e^{\left(\frac{\ln\left(\alpha_{u\_min}\right)\times iter}{max\_iter}\right)}$$

where max_iter is the number of maximum iterations, iter is the number of current iteration and $\alpha_{u\_min}$ is the minimum upper bound of the $\alpha_{r}$ in the final iteration. In this study, $\alpha_{u\_min}\;$= 0.3.

● Find a new location if the current movement cannot lead particle to a better point

**Figure 5:**
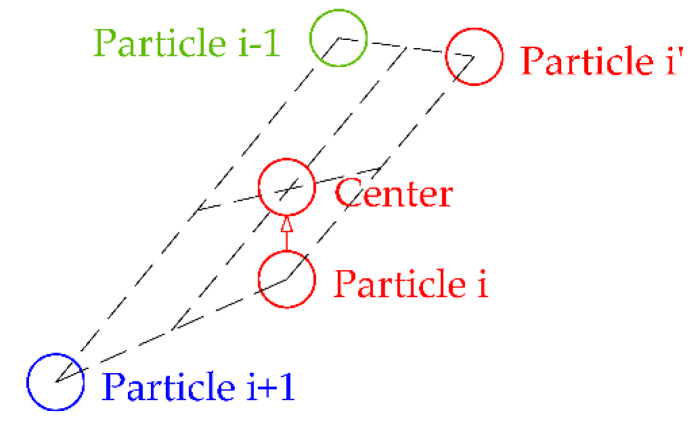
Graphical representation of location updating.

If the particle moves to a new place but get a worse function value, why should it move in that direction? It should find another new location. In order to fully utilize the information given by the particles *i*-1, *i*+1 and *i* and *i*’, it is recommended that the new location is the center of these four particles (Figure 5). The new location for particle *i* is written as follows. (Appendix A Algorithm A5, line 13)

(13) $$x_{i}^{’’}\;=\;\frac{1}{4}(x_{i-1}+x_{i+1}+x_{i}+x_{i}^{’})$$

● Do more random operations in early iterations to improve exploration

The original EM algorithm is somehow weak in exploration, and randomly generating of samples could help a lot in jumping out from local minimum. Therefore, in the early iterations, instead of doing the movement operation to the number of $n_{random}$ worst particles, they are randomly regenerated. After a certain iteration (called the transition iteration), only the worst particle is regenerated. Other particles do the normal attracting and repelling movement operations with their adjacent sorted partners. In this study, $n_{random}$ is 0.3 times the number of particles ($n_{particles}$), and the transition iteration is 0.1 times the number of maximum iterations (max_iter). In other words, $n_{random}=0.3\times n_{particles}\;$ from 1 to (0.1×max_iter) iterations, while $n_{random}$= 1 from (0.1×max_iter) to max_iter iterations. (Appendix A)

**Figure 6:**
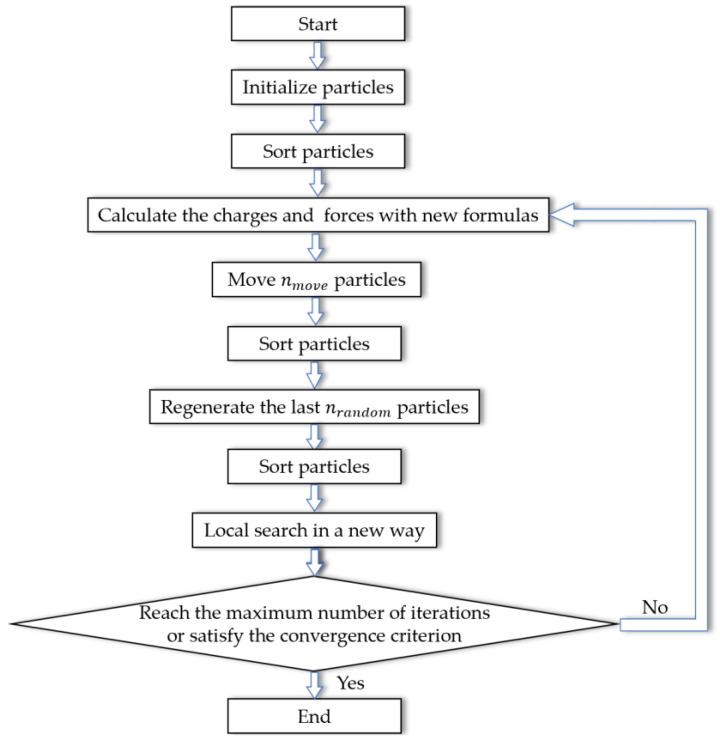
Flow chart of the modified EM algorithm.

The flow chart of the modified EM algorithm is shown in Figure 6. The complete pseudocode of the modified EM algorithm is shown in Appendix B.

## 4. Illustrative Examples

In this section, the modified EM algorithm is first tested in several benchmark functions, as compared with the original EM method [24] and particle swarm optimization (PSO) algorithm [39]. Then, the modified EM algorithm is applied and validated in a numerical truss model for model parameter identification, under different conditions of noise contamination and information incompleteness. Finally, an experimental shear-building model further validates the modified EM algorithm for structural model identification.

### 4.1. Benchmark Functions

In order to test the performance of the proposed modified EM method in numerical function optimization, numerical experiments are conducted on six well-known benchmark functions. Table 1 provides detailed information about these benchmark functions. Here, we compare the performance of three algorithms: the modified EM algorithm, the original EM algorithm and the PSO algorithm. In the numerical experiments, each algorithm is independently run 50 times for each benchmark function. The termination criterion of these three algorithms, i.e., maximum number of iterations, is set to 1000. The number of particles is set to 16 for the modified EM algorithm and the original EM algorithm. In the original EM algorithm, the local search parameter *δ* is set to 0.001. There is a built-in function for PSO algorithm implementation in MATLAB, and the control parameters are set as default values. The computing hardware of the numerical experiment was a laptop with Intel Core i7-8650U @ 1.90 GHz and 16 GB RAM, and the software environment was MATLAB R2019b on Windows 10 platform.

**Figure 7:**
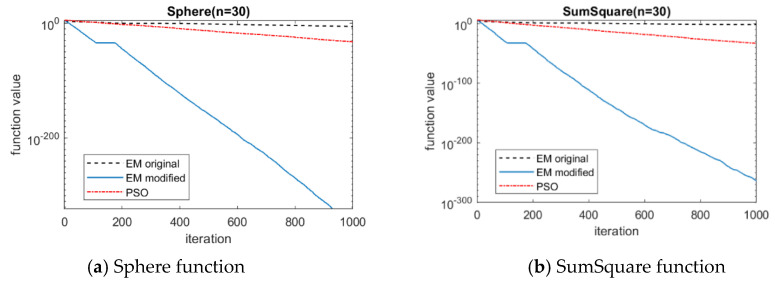

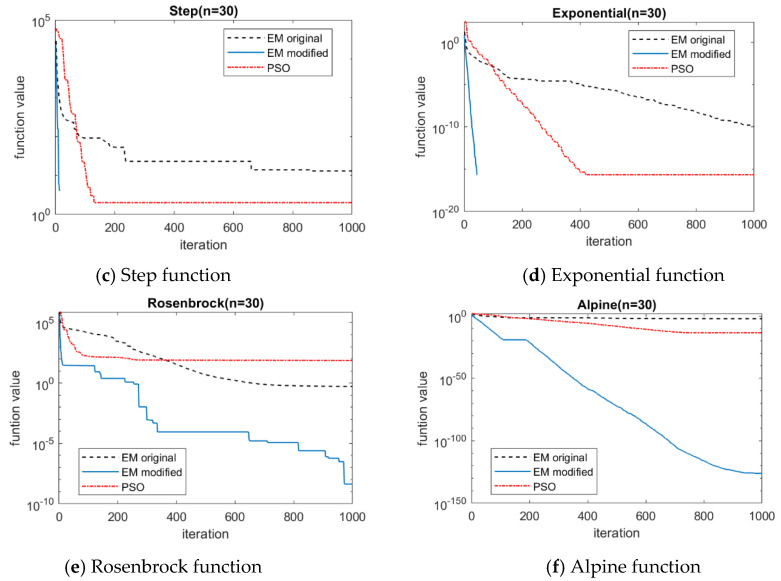
Convergence lines of six benchmark functions with 30 dimensions. (**a**): Sphere; (**b**): SumSquare; (**c**): Step; (**d**) Exponential; (**e**) Rosenbrock; (**f**) Alpine.

The mean, maximum and minimum values of the best solution and average computation time of six benchmark functions (*n* = 30) is tabulated in Table 2. It can be seen that the modified EM algorithm outperforms the original EM algorithm and the PSO algorithm in accuracy. For average computation time, the modified EM algorithm is much less than the original EM algorithm and is approximately the same level as the PSO algorithm. The convergence curves of six benchmark functions when using these three algorithms are shown in Figure 7. It can be seen that the modified EM algorithm has faster convergence rate than the original EM algorithm and the PSO algorithm. It is worth noting that for functions step and exponential, the convergence curves in the figure are suddenly cut off because their values drop to zero at very early iteration.

### 4.2. Numerical Truss Model

**Figure 8:**
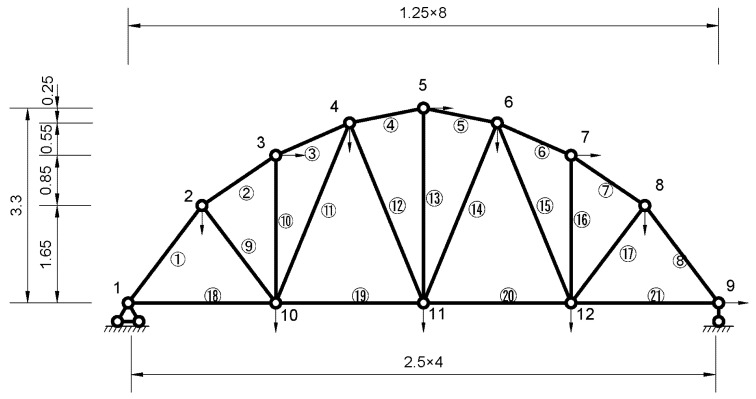
A 21-bar planar truss model (unit: m).

A simply supported truss model with 21 bars is considered in this example. The configuration and dimension of the truss model is shown in Figure 8. The truss has a span of 10 m and a height of 3.3 m. The elastic module of material is 1.8 GPa, the mass density of material is 2200 kg/m^3^ and the sectional area of all bars is 0.0025 m^2^. For finite element modeling of the truss, 12 nodes and 21 bar elements are used. For one planar bar element, there are two nodes, with two degrees of freedom (DOFs) for each node, i.e., one in horizontal direction and the other in vertical direction. Minus the boundary constraints (2 DOFs in node 1 and 1 DOF in node 9), there are 21 DOFs (2 × 12 − 3 = 21). It is assumed that there are some damages in elements 19 and 20, and the damage severity is −15% in element 19 and −20% in element 20. Overall, 21 stiffness parameters *θ_n_* (*n* = 1, 2, …, 21) were used to adjust the values of element stiffness *ke_n_*, so the updated ones would have the values of *ke_n_*(1+*θ_n_*) (*n* = 1, 2, …, 21), respectively.

In order to consider the noise contamination levels and information incompleteness of the modal data, four cases are assumed. For each case, it is assumed that 20 sets of modal data are available for each simulation, and the simulation are run independently 100 times. For complete measurement, the truss should have 21 DOFs to be measured and 21 modes to be available. In reality, the complete information cannot be achieved. For case 1, 2 and 4, it is assumed that there are 11 measurements (shown in Figure 8 with arrows on the nodes), while there are 7 measurements for case 3 (remove the measurements on nodes 2, 4, 6 and 8). Here, it is assumed that there are only first 8 modes for case 1, 2 and 3, while there are only first 5 modes for case 4. These four cases are listed as follows.

- Case 1: 1% noise for frequency, 5% noise for mode shape, 11 measurements and 8 modes.
- Case 2: 2% noise for frequency, 10% noise for mode shape, 11 measurements and 8 modes.
- Case 3: 1% noise for frequency, 5% noise for mode shape, 7 measurements and 8 modes.
- Case 4: 1% noise for frequency, 5% noise for mode shape, 11 measurements and 5 modes.

The statistics of identified stiffness parameters for elements 19 and 20 are shown in Table 3 after 100 simulations. It can be seen that the identified stiffness parameters agree well with the preset values for both four cases. The standard deviations are larger in case 2, 3 and 4 compared with case 1, especially for case 3. This phenomenon indicates that noise contamination and information incompleteness will cause more uncertainty in the identification results, especially for the measurement incompleteness.

**Figure 9:**
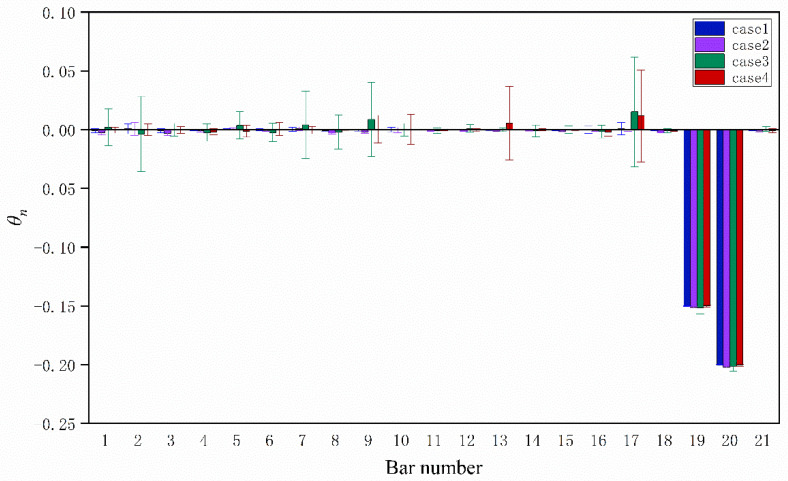
Updated stiffness parameters for truss model.

The statistics of identified stiffness parameters for all elements are shown in Figure 9 after 100 simulations, where the bars represent the mean values and I-shaped marks represent plus or minus one standard deviations with mean values. It can be seen that the mean values agree well with the preset values. Except for elements 19 and 20, the mean values of *θ*_n_ are almost zero. However, for case 3 and case 4, there are large standard deviations in some element stiffness parameters. This phenomenon indicates that information incompleteness has a great influence on the uncertainty of identification results. One may mitigate this effect by placing more sensors to collect measurement data.

### 4.3. Experimental Shear-Building Model

**Figure 10:**
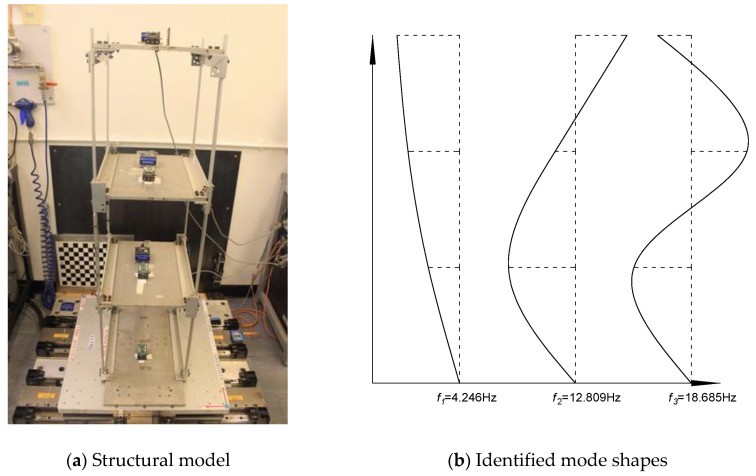
The tested three-storey shear-type structure model and its identified mode shapes. (**a**): Structural model; (**b**): Identified mode shapes.

A three-storey shear-building model [40,41,42] (Figure 10) was used to demonstrate the proposed technique. The dimensions of the model were 401 mm × 314 mm × 1158 mm (width × depth × height). The nominal values of storey mass were *m*_1_ = 5.63 kg, *m*_2_ = 6.03 kg and *m*_3_ = 4.66 kg. The nominal values of interstorey stiffness were *k*_1_ = 20.88 kN/m, *k*_2_ = 22.37 kN/m and *k*_3_ = 24.21 kN/m. The natural frequencies were then calculated as 4.5450, 13.023 and 18.210 Hz for the nominal model. In order to update the structural model, three stiffness parameters *θ*_1_, *θ*_2_ and *θ*_3_ were used to adjust the values of interstorey stiffness, so the updated ones would have the values of *k*_1_(1+*θ*_1_), *k*_2_(1+*θ*_2_) and *k*_3_(1+*θ*_3_), respectively. The structural model was fixed in a shake table and subjected to base excitations. The acceleration responses of all the three stories and the base were measured with a sampling frequency of 100 Hz. Specifically, 10 data sets with 60,000 points (10 min) for each data set were obtained.

The frequencies and mode shapes were identified using the frequency domain decomposition method [43]. The mean values for modal frequency are shown in Table 4, which are very close to the results identified by some other methods [41,42]. The identified mode shapes are shown in Figure 10. By using the proposed structural identification method, the identified stiffness parameters for *θ*_1_, *θ*_2_ and *θ*_3_ are −0.221, 0.099 and 0.032, respectively. The stiffness of the first storey has a large reduction, which may be due to the loosening of bolts used to fix the structural model. Table 4 shows the comparison of modal frequencies and MAC (Modal Assurance Criterion) values for experimental model, nominal model and updated model. It can be seen that the modal frequencies of the updated model are more close to those of the experimental model, and the same goes for the MAC values (the larger of MAC value, the similarity of the two mode shapes). Although there are still some differences in modal parameters, these are smaller than those between the nominal model and the experimental model.

## 5. Discussions

In this study, a modified EM algorithm is proposed to identify structural model parameters. The modified EM algorithm is more accurate than the original one, i.e., the convergence is faster and the calculation time is reduced. Compared with the PSO algorithm, it also has advantage in accuracy. Two application examples both show that the identification accuracy of the approach is very high, even in the case of large noise level and incomplete information. Nevertheless, the following points are worth for further study and discussion:

(1) For heuristic algorithms, due to its swarm and stochastic features, the solution results are often random in a small range. In order to eliminate the randomness, hybrid optimization algorithms are often used to combine the global searching ability of heuristic algorithm and the local precise searching ability of traditional algorithm, so as to achieve better solving performance.

(2) Due to the unavoidable measurement noise, the existence of model errors and incomplete information, the identified model parameters have certain uncertainties. How to quantify these uncertainties deserves further study.

(3) This study is based on the least squares principle for model parameter identification. If there is little information, the model may become unidentifiable, that is, there are multiple solutions. This situation may need to be treated by other methods such as Bayesian inference [44].

(4) The number and arrangement of sensors will have a certain impact on the identification accuracy. In general, the more the number of sensors, the more sufficient the information, and thus will improve the accuracy of identification. It is also necessary to optimize the arrangement of sensors. Different layouts of sensors will produce different identification accuracy for a certain parameter.

## 6. Conclusions

A modified EM optimization algorithm is proposed to identify structural parameters using modal data. The most important contribution of this study is the improvements of the original EM algorithm. Several modifications are made to the original EM algorithm to improve the optimization performance. A new local search strategy, new charge and force calculation formulas, new particle movement and updating rules are proposed to enhance the convergence and accuracy of the algorithm. The test results of six well-known benchmark functions show that the modified EM algorithm outperforms the original EM algorithm in both accuracy and efficiency and outperforms the popular PSO algorithm in accuracy.

The second contribution of this study is the successful introduction of the modified EM algorithm into the parameter identification of structural models, which has achieved good results. Two illustrative examples are presented to demonstrate the applicability in structural model identification. The identification results of the numerical truss model agree well with the preset values of model parameters, even under the condition of a high noise level of 10% and incomplete measurement of few DOFs and modes. The simulation results also show that the information incompleteness has a great influence on the uncertainty of identification results, and one may mitigate this effect by densely placement of sensors. The identification results of the experimental shear-building model further validate the effectiveness and reliability of the proposed algorithm for structural model identification. The modal parameters of the updated structural model are more close to those of the experimental model than before updating.

Furthermore, the modified EM algorithm is not only useful for the structural model identification but also helpful when EM algorithm is applied for other optimization problems. In addition, the proposed modifications can be inspiring for improving other heuristic algorithms. Still and all, expanding application field of proposed algorithm, hybrid optimization algorithms development and structural parameters uncertainty studies would be the directions for future research.

## Figures and Tables

**Table 1:** Test suite with six benchmark functions.

Name	Function	Search Range	Accepted Accuracy
Sphere	$f_{1}\left(X\right)=\overset{n}{\underset{i=1}{\sum }}x_{i}^{2}$	${\left[-100,\;100\right]}^{n}$	$1\times 10^{-8}$
SumSquare	$f_{2}\left(X\right)=\overset{n}{\underset{i=1}{\sum }}i\cdot x_{i}^{2}$	${\left[-100,\;100\right]}^{n}$	$1\times 10^{-8}$
Step	$f_{3}\left(X\right)=\overset{n}{\underset{i=1}{\sum }}{\left(x_{i}+0.5\right)}^{2}$	${\left[-100,\;100\right]}^{n}$	$1\times 10^{-8}$
Exponential	$f_{4}\left(X\right)=\exp(0.5\overset{n}{\underset{i=1}{\sum }}x_{i}^{2})-1$	${\left[-1.28,\;1.28\right]}^{n}$	$1\times 10^{-8}$
Rosenbrock	$f_{5}\left(X\right)=\overset{n-1}{\underset{i=1}{\sum }}\left[100{\left(x_{i+1}-x_{i}^{2}\right)}^{2}+{\left(x_{i}-1\right)}^{2}\right]$	${\left[-5,\;10\right]}^{n}$	$5\times 10^{0}$
Alpine	$f_{6}\left(X\right)=\overset{n}{\underset{i=1}{\sum }}\left|x_{i}\cdot \sin x_{i}+0.1\cdot x_{i}\right|$	${\left[-10,\;10\right]}^{n}$	$1\times 10^{-8}$

**Table 2:** Accuracy comparison on the six benchmark functions with dimension *n* = 30.

Function		Sphere	SumSquare	Step	Exponential	Rosenbrock	Alpine
Original EM	mean	1.20 × 10^−6^	0.0089	13.64	1.03 × 10^−10^	21.28	0.0031
	max	1.74 × 10^−6^	0.12	22	1.45 × 10^−10^	140.65	0.015
	min	6.89 × 10^−7^	2.93 × 10^−5^	6	6.66 × 10^−11^	7.33 × 10^−4^	5.32 × 10^−5^
	avg time	9.24 s	9.60 s	9.51 s	9.38 s	9.03 s	9.46 s
Modified EM	mean	0	7.10 × 10^−239^	0	0	0.0038	5.04 × 10^−107^
	max	0	3.55 × 10^−237^	0	0	0.053	2.52 × 10^−105^
	min	0	5.08 × 10^−241^	0	0	1.15 × 10^−9^	2.79 × 10^−141^
	avg time	2.78 s	2.88 s	4.89 s	2.65 s	3.66 s	3.01 s
PSO	mean	2.93 × 10^−34^	2.25 × 10^−29^	2.68	4.22 × 10^−16^	30.19	1.07 × 10^−14^
	max	7.04 × 10^−33^	1.12 × 10^−27^	19	1.78 × 10^−15^	138.04	3.86 × 10^−14^
	min	2.02 × 10^−37^	6.76× 10^−37^	0	2.22 × 10^−16^	0.21	1.33 × 10^−15^
	avg time	3.10 s	3.24 s	2.51 s	2.71 s	2.96 s	2.97 s

**Table 3:** The identified results of the model parameters.

	Case 1		Case 2		Case 3		Case 4	
	Mean	Standard Deviation	Mean	Standard Deviation	Mean	Standard Deviation	Mean	Standard Deviation
*θ_19_*	−0.1502	2.401 × 10^−4^	−0.1510	4.082 × 10^−4^	−0.1515	5.380 × 10^−3^	−0.1501	7.174 × 10^−4^
*θ_20_*	−0.2006	6.237 × 10^−4^	−0.2021	6.321 × 10^−4^	−0.2013	4.450 × 10^−3^	−2.005	7.855 × 10^−4^

**Table 4:** Comparison of modal parameters of nominal and updated models with those of experimental model.

Mode	Identified Frequency	Nominal Frequency	MAC_n−e_	Updated Frequency	MAC_u−e_
1	4.246	4.545 (7%)	0.9975	4.258 (0.3%)	0.9997
2	12.809	13.023 (1.6%)	0.9842	12.783 (0.2%)	0.9949
3	18.685	18.21 (−2.5%)	0.9589	18.607 (0.4%)	0.9698

Notes: MAC_n−e_ and MAC_u−e_ are the MAC values of mode shapes between nominal model and experimental model and between updated model and experimental model, respectively.

## Nomenclature

The Nomenclature is listed as follows.

$a_{j,i}$	A scaling factor of the predicted mode shape ${\hat{\phi }}_{j}\left(\theta \right)$ with respect to $\phi_{j,i}$
DOFs	Degrees of freedom
f(·)	Objective function to be minimized
$F_{a}$	Attraction force
$F_{r}$	Repulsion force
$F_{i}$	The resultant force on particle *i* of the original EM algorithm
$f_{j,i}$	The measured modal frequency of *j*^th^ mode in the *i*^th^ data set
${\hat{f}}_{j}$	The predicted modal frequency of *j*^th^ mode
***M***	Global mass matrix
MAC	Modal Assurance Criterion
*m*	The number of particles in the original EM algorithm
*max_iter*	The number of maximum iterations
*N*	The number of DOFs
n	The dimension of the problem
$n_{particles}$	The number of particles
$n_{random}$	The number of particles regenerated randomly in each iteration
*N*_θ_	The number of parameters
*N*_e_	The total number of finite elements
*N*_m_	The number of modes
*N*_s_	The number of data sets of measured modal data
$q_{i}$	The charge quantity of particle *i*
***K***	Global stiffness matrix
*ke*_i_	The stiffness matrix of the *i*^th^ element
*RNG*	The feasible step size vector
$v_{EM}$	Movement vector
$x_{i}$	Particle *i*
$x_{best}$	The particle with the optimal objective function value
$x_{2^{nd}best}$	The particle with the second best objective function value
$x_{3^{rd}best}$	The particle with the third best objective function value
$\alpha_{r}$	Rate of repulsion uniformly distributed in $\left[0,\alpha_{u}\right]$
$\alpha_{u}$	Upper bound of $\alpha_{r}$ in each iteration
δ	Step size in the local search of the original EM algorithm
***θ***	Parameters of the stiffness matrix ***K***
λ	The random step of movement used in the original EM algorithm
$\phi_{j,i}$	The measured mode shape of *j*^th^ mode in the *i*^th^ data set
${\hat{\phi }}_{j}$	The predicted mode shape of *j*^th^ mode

## Appendix A

The pseudocodes of the improvements presented in Section 3.2 are given below.

A1. Initialize

**Algorithm** **A1.**$\left[Particles,dim,n_{move}\right]=Initialize\left(n_{particles},n_{random},L,U\right)$
1: dim←length(L) % U and L are row vectors 2: $n_{move}\leftarrow n_{particles}-n_{random}$ 3: $Particles\leftarrow ones\left(n_{parricles},1\right)*L+rand\left(n_{particles},dim\right).*\left(U-L\right)$ 4: **for** i=1 to n_particles **do** 5: f_vals(i)=f(Particles(i,:)) % *f_vals* is a column vector 6: **end for** 7: Particles←[Particles,f_vals] % $Particles\left(i,:\right)=\left[x_{i}\;,\;\;f\left(x_{i}\right)\right]$ 8: Particles←Sort_particles(Particles,dim)

A2. Sort particles

**Algorithm** **A2.**Particles=Sort_particles(Particles,dim)
1: Particles←sortrows(Particles,dim+1) % $Particles\left(i,:\right)=\left[x_{i}\;,\;\;f\left(x_{i}\right)\right]$

A3. Check boundary

**Algorithm** **A3.**$P_{temp}=Check\_boundary\left(P_{temp},L,U\right)$
1: $P_{temp}\left(P_{temp}>U\right)\leftarrow U\left(P_{temp}>U\right)$ % $P_{temp}\left(i\right)\leftarrow U\left(i\right)$ if $P_{temp}\left(i\right)>U\left(i\right)$ 2: $P_{temp}\left(P_{temp}<L\right)\leftarrow L\left(P_{temp}<L\right)\;\;$ % $P_{temp}\left(i\right)\leftarrow L\left(i\right)$ if $P_{temp}\left(i\right)<L\left(i\right)$

A4. Charge calculation

**Algorithm** **A4.**$q=Cal\_q\left(Particles,n_{move}\right)$
1: **for** $i\;=\;1\;to\;n_{move}+1$ **do** 2: $q\left(i\right)\leftarrow \left(f\left(x_{i}\right)-f\left(x_{1}\right)\right)/\left(f\left(x_{n_{move}+1}\right)-f\left(x_{1}\right)\right)\;\;$% $Particles\left(i,:\right)=\left[x_{i}\;,\;\;f\left(x_{i}\right)\right]$ 3: **end for**

A5. Move $n_{move}$ particles

**Algorithm** **A5.** $Particles=Move\left(f,\alpha_{u},Particles,q,n_{move},dim,L,U\right)$
1: $F_{r}\leftarrow q\left(2\right)\left(x_{1}-x_{2}\right)$ 2: $\alpha_{r}\leftarrow rand*\alpha_{u}$ 3: $P_{temp}\leftarrow x_{1}+\alpha_{r}F_{r}$ 4: $P_{temp}\leftarrow Check\_boundary\left(P_{temp},L,\;U\right)$ 5: $Particles\left(1,:\right)\leftarrow \left[P_{temp},\;\;f\left(P_{temp}\right)\right]$ % $x_{1}\leftarrow P_{temp}$ 6: **for** $i\;=\;2\;ton_{move}$ **do** 7: $\alpha_{r}\leftarrow rand*\alpha_{u}$ 8: $F_{a}\leftarrow q\left(i-1\right)q\left(i\right)\left(x_{i-1}-x_{i}\right)$ 9: $F_{r}\leftarrow q\left(i+1\right)q\left(i\right)\left(x_{i}-x_{i+1}\right)$ 10: $P_{temp}\leftarrow x_{i}+\left(1-\alpha_{r}\right)F_{a}+\alpha_{r}F_{r}$ 11: $P_{temp}\leftarrow Check\_boundary\left(P_{temp},L,U\right)$ 12: **if** $Particles\left(i,end\right)<f\left(P_{temp}\right)$ 13: $P_{temp}\leftarrow \left(P_{temp}+\overset{j=i+1}{\underset{j=i-1}{\sum }}Particles\left(j,1:dim\right)\right)/4\;\;$% Center of four particles 14: **end if** 15: $Particles\left(i,:\right)\leftarrow \left[P_{temp},\;\;f\left(P_{temp}\right)\right]$ % $x_{i}\leftarrow P_{temp}$ 16: **end for**

A6. Randomly regenerate the last $n_{random}$ particles

**Algorithm** **A6.** $Particles=Random\_regenerarte\left(f,Particles,\;n_{move},L,U\right)$
1: **for** $i\;=\;n_{move}+1\;to\;n_{particles}$ **do** 2: $x_{i}\leftarrow rand\left(1,\;n\right).*\left(U\;-\;L\right)\;+\;L$ 3: $Particles\left(i,:\right)=\left[x_{i},\;f\left(x_{i}\right)\right]$ 4: **end for**

A7. Local search

**Algorithm** **A7.** $Particles=Local\_search\left(f,Particles,n_{branch},n_{leaf},dim,L,U\right)$
1: $LS\_radius\leftarrow x_{1}-x_{2}\;\;$ % or $LS\_radius\leftarrow \|x_{1}-x_{random\_integer\left(2,3\right)\|}\;$ 2: **for** $i\;=\;1\;to\;n_{branch}$ **do** 3: $P\_LS\_branch_{i}\leftarrow x_{1}+LS_{radius}*\left(rand\left(1,dim\right)-0.5\right)$ 4: $P\_LS\_branch_{i}\leftarrow Check\_boundary\left(P\_LS\_branch_{i},L,U\right)$ 5: **if** $f\left(P\_LS\_branch_{i}\right)<Particles\left(i,end\right)$ % Particles(i,end) is $f\left(x_{i}\right)$ 6: Particles(2,:)← Particles(1,:) % $x_{2}\leftarrow x_{1}$ 7: $Particles\left(1,:\right)\leftarrow \left[P\_LS\_branch_{i},f\left(P\_LS\_branch_{i}\right)\right]$ % $x_{1}\leftarrow P\_LS\_branch_{i}$ 8: **else if** $f\left(P\_LS\_brahch_{i}\right)<f\left(x_{2}\right)$ 9: $Particles\left(2,:\right)\leftarrow \left[P\_LS\_branch_{i},f\left(P\_LS\_branch_{i}\right)\right]$ % $x_{2}\leftarrow P\_LS\_branch_{i}$ 10: **end if** 11: **for** $k\;=\;1\;to\;n_{leaf}$ **do** 12: $P\_LS\_leaf_{k}\leftarrow P\_LS\_brahch_{i}+LS\_radius*\left(\mathrm{rand}\left(1,\;\dim\right)–0.5\right)$ 13: $P\_LS\_leaf_{k}\leftarrow Check\_boundary\left(P\_LS\_leaf_{k},L,U\right)$ 14: **if** $f\left(P\_LS\_leaf_{k}\right)<f\left(x_{1}\right)$ 15: Particles(2,:)← Particles(1,:) %$\;\;x_{2}\leftarrow x_{1}$ 16: $Particles\left(1,:\right)\leftarrow \left[P\_LS\_leaf_{k},f\left(P\_LS\_leaf_{k}\right)\right]\;\;$ % $x_{1}\leftarrow P\_LS\_leaf_{k}$ 17: break 18: **elseif** $f\left(P\_LS\_leaf_{k}\right)<f\left(x_{2}\right)$ 19: $Particles\left(2,:\right)\leftarrow \left[P\_LS\_leaf_{k},f\left(P\_LS\_leaf_{k}\right)\right]$ % $x_{2}\leftarrow P\_LS\_leaf_{k}$ 20: **end if** 21: **end for** 22: **end for**

## Appendix B

The complete pseudocode of the modified EM algorithm is presented below.

**Algorithm****A8.** Complete pseudocode $\left[x_{best},f\left(x_{best}\right)\right]\;=\;EM\_m\left(f\;,n_{particles},L,U,max\_iter\right)$
1: $\;set\_parameters\left(\alpha_{u},\alpha_{u\_min}\;\;,n_{random},rand\_iter,n_{branch},n_{leaf}\right)$ 2: $\left[Particles,dim,n_{move}\right]\leftarrow Initialize\left(n_{particles},n_{random},L,U\right)$ 3: **for** iter= 1 to MAX_iter **do** 4: $q\leftarrow Cal\_q\left(Particles,n_{move}\right)$ 5: $Particles\leftarrow Move\left(f,\alpha_{u},Particles,n_{move},dim,L,U\right)$ 6: Particles←Sort_particles(Particles,dim) 7: $Particles\leftarrow Random\_regenerarte\left(f,Particles,\;n_{move},L,U\right)$ 8: Particles←Sort_particles(Particles,dim) 9: $Particles\leftarrow Local\_search\left(f,Particles,n_{branch},n_{leaf},dim,L,U\right)$ 10: $\alpha_{u}\leftarrow \exp\left(\ln\left(\alpha_{u\_min}\right)*iter/\max\_iter\right)$ 11: **if** iter==rand_iter 12: $n_{move}\leftarrow n_{particles}-1$ 13: $n_{random}\leftarrow 1$ 14: **end if** 15: **end for** 16: $\left[x_{best},f\left(x_{best}\right)\right]\leftarrow \left[Particles\left(i,1:dim\right),Particles\left(1,end\right)\right]$
